# Sonoporation by low-frequency and low-power ultrasound enhances chemotherapeutic efficacy in prostate cancer cells *in vitro*

**DOI:** 10.3892/ol.2013.1389

**Published:** 2013-06-10

**Authors:** YU WANG, WEN-KUN BAI, E. SHEN, BING HU

**Affiliations:** Department of Ultrasound in Medicine, Shanghai Jiao Tong University Affiliated Sixth People’s Hospital, Shanghai Institute of Ultrasound in Medicine, Shanghai 200233, P.R. China

**Keywords:** low-frequency ultrasound, low-energy ultrasound, microbubble, mitoxantrone delivery

## Abstract

Combination therapy is used to optimize anticancer efficacy and reduce the toxicity and side-effects of drugs upon systemic administration. Ultrasound (US) combined with micro-bubbles (UM) enhances the intracellular uptake of cytotoxic drugs by tumor cells, particularly drug-resistant cells. In the present study, low-frequency and low-energy US (US irradiation conditions: frequency, 21 kHz; power density, 0.113 W/cm^2^; exposure time, 2 min at a duty cycle of 70%; and valid treatment time, 84 sec) were used in combination with microbubbles (100 *μ*l/ml) to deliver mitoxantrone HCl (MIT) to DU145 cells. The results showed that UM did not change the cell viability in the short- or long-term. However, UM statistically enhanced the therapeutic effects and up to 31.26±3.34% of the cells exposed to UM were permeabilized compared with 9.74±2.55% of cells in the control, when using calcein (MW, 622.53) as a fluorogenic marker. Notably, UM affected the migration capability of the DU145 cells at 6 h post-treatment. In conclusion, the ultrasonic parameters used in the present study enhanced the chemotherapeutic effect and reduced the unwanted side-effects of MIT.

## Introduction

As an established therapeutic method, ultrasound (US) is used for bone fracture healing, hyperthermia and the ablation of solid tumors (1). Furthermore, in this newly emerging field, US-mediated microbubble destruction, a noninvasive approach, has been shown to possess significant potential to increase the permeability of cell membranes and tissues to various substances. Since US-mediated microbubble destruction is able to reversibly disrupt biological barriers, particularly cell membranes, large quantities of molecules may then be delivered into tumor cells, particularly drug-resistant cells. The mechanism by which this occurs is considered to be sonoporation, resulting from oscillations of the gas bubbles in the media, which cause cavitation close to the cell surface and subsequent membrane disruption that allows increased drug internalization (2). It has been demonstrated that intracellular uptake is greatly enhanced by diagnostic microbubbles used for US imaging (3–5). At particular ultrasonic frequencies, microbubbles have been shown to greatly enhance transient sonoporation (6). These microbubbles, oscillating in the presence of US, create localized shear stress or ‘microstreaming’ or they may expand and collapse (‘transient cavitation’) to create intense local heating and pressure (7). This type of transient cavitation effect is considered to occur more at low frequencies (8). Schlicher *et al* demonstrated transient pores (<28 nm diameter) in the plasma membrane of cells, following exposure to low frequency US (24 kHz) (9).

Prostate cancer (PCa) is one of the most common types of cancer among the male population of Western countries, second only to skin cancer (10,11). Hormonal therapy allows long-lasting and effective control of cancer-related symptoms at advanced stages. However, in almost all patients with metastatic PCa, the disease progresses when it becomes castration-resistant (CRPC) (12). At that stage, second-line endocrinal therapy and chemotherapy should be administered. In order to maintain sufficient doses of chemotherapeutic drugs in the cancerous tissue, all tissues are exposed to various concentrations of cytotoxic drugs during systemic administration. Combination therapy is used to optimize anticancer efficacy and reduce the toxicity and side-effects of drugs upon systemic administration. US combined with microbubbles (UM) is able to enhance the intracellular uptake of the cytotoxic drugs by the tumor cells, particularly the drug-resistant cells.

Sonoporation, electroporation, microinjection and laser irradiation are all able to enhance the transmembrane delivery of therapeutic molecules. However, sonoporation is considered to be a ‘gentle’ technique. Non-inertial cavitation is generated by the alternate growth and shrinkage caused by contrast agents (CAs) oscillating, which occurs at low acoustic pressures. Further complex non-linear interactions appear when the ultrasonic pressure reaches a certain threshold, and at that time the microbubble explodes and pores form, which enhances the intracellular uptake or kills the cells. Overall, inertial cavitation occurs at relatively high pressure amplitudes and CAs contract and collapse ‘violently’ (13). Khanna *et al*, who performed the first notable study, used US waves to make blood cells release hemoglobin (14). The study into US waves by Kinoshita and Hynynen showed that increasing sonoporation typically lessened cellular viability (15). However, Rodamporn *et al* concluded that improved conditions reduced the loss of viability while maintaining high transfection rates (16). To the best of our knowledge, the present study is the first to state that low-frequency and low-energy US are able to reduce the loss of viability while maintaining high sonoporation in DU145 PCa cells.

## Materials and methods

### Cell culture

DU145 cells, a human PCa cell line, were obtained from the Cell Bank of the Chinese Academy of Sciences (Shanghai, China) and used to study the chemotherapy response at the cellular level. The cells were maintained in Dulbecco’s modified Eagles medium (DMEM; Gibco, Grand Island, NY, USA) supplemented with 10% heat-inactivated fetal bovine serum (FBS; Invitrogen, Carlsbad, CA, USA) in 5% CO_2_ humidified air at 37°C. This study was approved by the ethics committee of Shanghai Jiao Tong University Affiliated Sixth People’s Hospital (Shanghai, China).

### Drugs

Mitoxantrone HCl (MIT; Sigma Aldrich, St. Louis, MO, USA) was dissolved in phosphate-buffered saline (PBS) at a concentration of 10 nM and the solution was stored at −20°C until use. MIT, which was approved by the FDA for hormone refractory PCa (HRPC) as a palliative treatment in 1996, is a cell cycle nonspecific agent that inhibits nucleic acid synthesis, leading to cell death. The dose limiting toxic effects are bone marrow suppression, leukopenia and thrombocytopenia.

### US apparatus

FS-450 ultrasonic processing (Shanghai Institute of Ultrasound in Medicine, Shanghai, China) with a SonoVue™ microbubble echo-contrast agent (Bracco SpA, Milan, Italy) was employed as previously described (17). The US irradiation conditions were as follows: frequency, 21 kHz; power density, 0.113 W/cm^2^; instrument exposure time, 2 min at a duty cycle of 70% (i.e., 7 sec ‘on’ time and 3 sec ‘off’ time); and a valid treatment time of 84 sec.

### Treatment

The cell suspensions were divided into four treatment groups: Group A, non-treated (control); group B, UM treatment; group C, MIT treatment; and group D, combined treatment with MIT and UM (MIT+UM). DU145 cells (1×10^6^ in 1 ml of medium) were transferred into 1.5-ml polystyrene test tubes, the diameter of which was the same as the probe being used. MIT (10 nmol) was added to groups C and D.

### Analysis of cell proliferation

Immediately following exposure to US, the cells were added to a 96-well plate at a density of 5,000 cells/well and incubated with 5% CO_2_ at 37°C. Subsequent to 24 h, 50 *μ*l dilution medium containing 10 *μ*l MTT solution (5 mg/ml) was used to replace the medium, and the plate was incubated for another 4 h until the liquid was removed. The formazan crystals that formed were dissolved with 150 *μ*l DMSO. Following agitation for 5 min, the absorbance of each well at a test wavelength of 570 nm was measured with a microculture plate reader (Bio-Tek, Winooski, VT, USA). The percentage cell viability was calculated as OD_exposed_ / OD_control_ × 100.

### Clonogenic assay

The long-term proliferation rate of the treated cells was measured by plate clonogenic assays. Subsequent to therapy, 200 cells/well were seeded into a 12-well plate, then incubated for two weeks to form colonies prior to fixation in 70% ethanol, staining with crystal violet and counting.

### Cell migration assay

The transwell apparatus (Costar, Cambridge, MA, USA) was used for the cell migration assays. There were 1×10^4^ cells in 100 *μ*l DMEM without FBS in the the upper polycarbonate membrane insert (pore size, 8 *μ*m), which was precoated with 24 mg/ml Matrigel (R&D Systems, Minneapolis, MN, USA), and there was 600 ml DMEM with 10% FBS in the lower chamber.

Following incubation for 8 h at 37°C in a 5% CO_2_ atmosphere, the upper cells were removed with a cotton swab and the cells adhering to the lower surface were fixed with 95% alcohol for 15–20 min and stained with crystal violet for 15 min. Finally, the total number of migratory cells was counted with a microscope.

### Analysis of DU145 cell permeability using calcein with or without UM

Calcein was used as the permeability tracer to further support our hypothesis that UM increases the absorption of drugs. Calcein (1 *μ*l, 25 mg/ml; Sigma Aldrich) was added to each sample just prior to exposure. Following incubation for 1 h at 37°C, the samples were washed three times with PBS, then 10,000 cells/sample were detected by flow cytometry using the CellQuest Pro software program (BD Biosciences, Franklin Lakes, NJ, USA) with an excitation wavelength of 492 nm and an emission wavelength of 518 nm (18). The results are expressed as the percentage of positive cells and fluorescence intensity with regard to the whole cell population, including any dead cells.

### Statistical analysis

Data are expressed as the mean ± SEM. The differences among the groups were analyzed using Student’s t-test or one-way ANOVA. P<0.05 was considered to indicate a statistically significant difference.

## Results

### Increasing the efficacy of chemotherapy (tests of cell proliferation)

**Figure 1.**
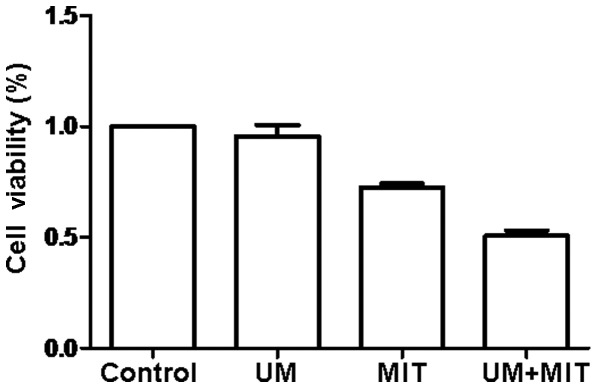
MTT assay showing the cell viability of each group at 24 h post-treatment (n=3). There was no significant effect on cell viability with UM alone. The viability of cells exposed to MIT+UM was significantly decreased (50.7%) compared with those exposed to MIT alone (72.3%; P<0.05), indicating that UM enhanced the chemotherapeutic efficacy of MIT in the DU145 cell line (P<0.05). Bars represent SEM. UM, ultrasound in combination with microbubbles; MIT, mitoxantrone HCl.

In order to examine the hypothesis that UM is able to enhance the efficacy of chemotherapy, the cytotoxicity on each group of the cells from the PCa cell line DU145 was evaluated using the MTT assay. Fig. 1 shows the results following incubation for 24 h, which indicated that the MIT+UM group had clear cytotoxicity compared with the other three groups. As shown in Fig. 1, compared with the controls, the cell viability of the UM group did not decrease (P>0.05), although the cell viability of the MIT (72.3%) and MIT+UM (50.7%) groups was significantly decreased (P<0.05). Compared with the MIT group, the cell viability of the MIT+UM group was significantly decreased (P<0.05).

### Viability of the reversibly permeabilized cells

**Figure 2.**
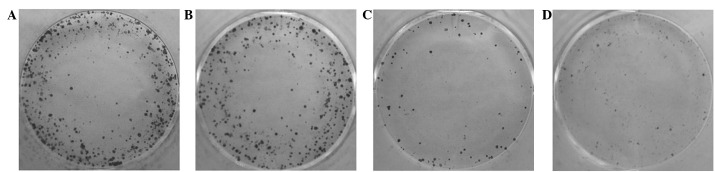
Images of the colony formation of the various groups. (A) Control group; (B) group administered with MIT; (C) group administered with UM; (D) group administered with MIT+UM. The DU145 cells fixed in 70% ethanol were stained with crystal violet. The clonogenicity of the cells exposed to UM was lower compared with the untreated cells and the numbers of each were 95.7±7.1 and 102.0±6.6 colonies, respectively (P>0.05). By contrast, the cells exposed to MIT and MIT combined with UM maintained their colony forming ability, with 45.0±6.0 and 18.7±5.5 colonies, respectively (P<0.05). Crystal violet staining. UM, ultrasound in combination with microbubbles; MIT, mitoxantrone HCl.

The long-term effects of MIT and UM, alone or in combination, on the DU145 cells were evaluated with the colony formation assay. The numbers of cell colonies formed are shown in Fig. 2. The clonogenicity of the cells exposed to UM (95.7±7.1) was lower compared with the untreated cells (102.0±6.6) (Fig. 2). By contrast, the cells exposed to MIT (45.0±6.0) and MIT combined with UM (18.7±5.5) had significantly lower colony forming numbers (P<0.05).

### Effects on the migration of the DU145 cells

**Figure 3.**
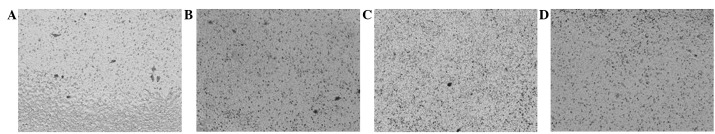
Representative cell migration numbers in human prostate cancer cells. Migration in the (A) control; (B) UM-treated; (C) MIT-treated; and (D) MIT+UM-treated groups. At 6 h post-treatment, UM, MIT and UM+MIT decreased the migration ability of the DU145 cells (P<0.05). The addition of UM resulted in significant suppression of DU145 migration compared with MIT alone, by 2.7±2.5 and 17.7±7.1, respectively (P<0.05), as compared with the control (75.7±7.5) and UM (45.3±6.5) groups. Crystal violet staining. UM, ultrasound in combination with microbubbles; MIT, mitoxantrone HCl.

The cell migration ability was assayed using the transwell apparatus in order to investigate the long-term biological effects of each treatment on the DU145 cells. The transmembrane cells of each group are shown in Fig. 3. Compared with the control group, the invasive ability of the DU145 cells in the UM (45.3±6.5), MIT (17.7±7.1) and MIT+UM (2.7±2.5) groups was significantly decreased (all P<0.05; Fig. 3). The results indicated that UM may enhance the ability of MIT in decreasing the colony forming ability of cells.

### Analysis of sonoporation efficacy

**Figure 4.**
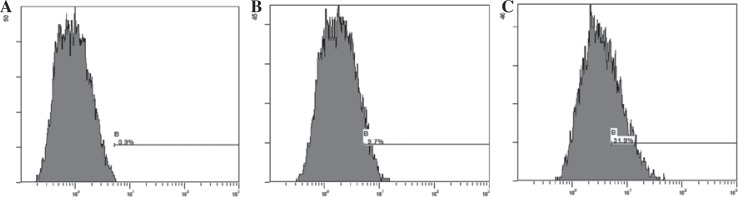
The mean percentage of fluorescence-positive cells in control at 1 h was 9.74±2.55 vs. 31.26±3.34% in cells exposed to UM (P<0.05). UM, ultrasound in combination with microbubbles. A, blank control group; B, ultrasound; C, UM.

Fig. 4 shows the percentage of intracellular calcein (a molecular fluorescent probe; MW, 622.53) induced by sonoporation. The cells treated with UM (31.26±3.34%) showed an increased percentage of fluorescence-positive cells compared with the control cells (9.74±2.55%) following incubation for 1 h (P<0.05).

## Discussion

Numerous side-effects follow effective MIT therapy. Combination therapy is used to optimize anticancer efficacy and reduce the toxicity and side-effects of drugs upon systemic administration. With its noninvasive and steerable nature, US is a useful tool in combined treatment with anti-cancer agents. Sonoporation has been associated with enhanced drug delivery in chemotherapy in primary studies concerning cutaneous melanoma (19,20) lymphoma (21) and oral cancer (22,23). It appears that there have been few studies focusing on PCa and combination therapy, and only a small number on the enhancement of permeability *in vivo* (24).

The present study is the first to investigate this strategy in PCa. The results revealed the successful enhancement of transmembrane MIT transport with the aid of UM, while simultaneously avoiding significantly affecting cell viability. This low frequency and low power-based method did not allow the microbubbles to make contact with the cell membrane immediately and did not exert significant additional stress on the cell membrane to cause death-induced pores. Notably, the study demonstrated for the first time that combining these strategies facilitated the uptake of MIT by DU145 cells in a number of ways. This alternative low-cost device may offer some quality of life improvement for patients, rather than other more expensive techniques, which may be welcomed by individuals in low-level income brackets. Further studies to gain a greater understanding of the mechanisms should be performed prior to this therapeutic method being used in clinical practice, to identify the most appropriate UM parameters that increase the efficacy of all types of therapeutic agents *in vivo* and are safe for normal tissues. The present study represents a first step towards combination therapy for PCa.
